# Severe-intensity constant-work-rate cycling indicates that ramp incremental cycling underestimates ⩒o_2max_ in a heterogeneous cohort of sedentary individuals

**DOI:** 10.1371/journal.pone.0235567

**Published:** 2020-07-06

**Authors:** Avigdor D. Arad, Kaitlyn Bishop, Deena Adimoolam, Jeanine B. Albu, Fred J. DiMenna

**Affiliations:** 1 Division of Endocrinology, Diabetes and Bone, Department of Medicine, Icahn School of Medicine at Mount Sinai, New York, NY, United States of America; 2 Department of Biobehavioral Sciences, Teachers College, Columbia University, New York, NY, United States of America; University of Bourgogne France Comté, FRANCE

## Abstract

In the absence of a ⩒o_2_-work-rate plateau, debate continues regarding the best way to verify that the peak ⩒o_2_ achieved during incremental exercise (⩒o_2peak_) is the “true ⩒o_2max_.” Oft-used “secondary criteria” have been questioned in conjunction with the contention that a severe-intensity constant-work-rate “verification bout” should be considered the “gold standard.” The purpose of this study was to compare the ⩒o_2peak_ during ramp incremental cycling (RAMP-INC) by a heterogeneous (with respect to body composition and sex) cohort of sedentary individuals with the ⩒o_2peak_ during severe-intensity constant-work-rate cycling (CWR) performed after RAMP-INC at the highest work rate achieved. A secondary purpose was to determine the degree to which traditional and newly-proposed age-dependent secondary criteria (RER, HR) identified RAMP-INC which CWR confirmed were characterized by a submaximal ⩒o_2peak_. Thirty-five healthy male (*n* = 19: 33.4 ± 6.3 yrs) and female (26.8 ± 3.6 yrs) sedentary participants performed RAMP-INC followed by CWR. The ⩒o_2peak_ values from the two tests were correlated (*r* = 0.96; *p* < 0.01; mean CV = 24%); however, ⩒o_2peak_ for CWR was significantly greater (29.6 ± 7.2 *v*. 28.6 ± 6.8 mL∙min^-1^∙kg^-1^; *p <* 0.01) with a mean bias of 0.98 mL∙min^-1^∙kg^-1^ (*z* = -2.9, *p* < 0.01). Both traditional and newly-proposed criterion values for RER were achieved during RAMP-INC by 33 of 35 participants (including 21 of 23 who registered a higher ⩒o_2peak_ on CWR). The traditional HR criterion value was achieved on only seven tests (three of which were confirmed to be characterized by a submaximal ⩒o_2peak_) while use of less stringent newly-proposed criteria resulted in acceptance of an additional seven tests of which five were confirmed to be submaximal. Severe-intensity CWR to limit of tolerance indicates that RAMP-INC underestimates ⩒o_2max_ in sedentary individuals and both traditional and newly-proposed secondary criteria are ineffective for identifying such tests.

## Introduction

The maximum rate of oxygen uptake (⩒o_2max_) is universally recognized as the criterion measure of cardiorespiratory fitness. Assessment of ⩒o_2max_, therefore, comprises a standard component of health/fitness testing in an array of settings spanning from elite-athletic to clinical. Early studies that established the concept required completion of a series of exhaustive exercise bouts at increasing constant work rates performed on separate days [1] or the same day [2]. The objective was to identify a range of work rates characterized by a similar peak ⩒o_2_ (⩒o_2peak_) response (a “⩒o_2_ plateau”) indicating an upper limit that could not be exceeded. As measurement technology improved and the need for time-efficient testing was recognized, single tests involving a progressive increase in work rate (incremental test; INC) either in steps [3] or continuously as a “smooth function of time” [4] (STEP-INC or RAMP-INC, respectively) became the standard methods of assessment. In theory, the continuous work-rate increase which characterizes RAMP-INC should be best suited for eliciting a ⩒o_2_ plateau because an extended stage does not have to be completed before a subsequent increase in work rate occurs. However, even when maximal effort appears present at exercise completion, a definitive plateau is often absent at limit of exercise tolerance (T_lim_) during RAMP-INC tests [5].

Given the importance placed on ⩒o_2max_ and the array of individuals that are critically assessed using its measurement, it is intuitive to suggest that the ⩒o_2peak_ achieved on a given INC should be verified as ⩒o_2max_ in some other way when a ⩒o_2_ plateau is absent. Controversy surrounds methods that have been employed for this purpose. One option is to extend the testing session with a constant-work-rate “verification bout” (CWR) performed at a work rate situated in the “severe-intensity domain” within which attainment of ⩒o_2max_ is an inevitable consequence of exercise maintained to T_lim_ [6]. The ⩒o_2peak_ recorded during a severe-intensity CWR bout can be used to test for the presence of a plateau [7, 8] as long as the work rate can be sustained long enough for ⩒o_2max_ to be reached; i.e., is not situated in the extreme-intensity domain [9]. However, Murias et al. (2018) recently argued that verification bouts are not necessary because they do not reveal a significantly different ⩒o_2peak_ compared to RAMP-INC for recreationally-active younger and older males [10]. They also reason that in contrast to indicating that the ⩒o_2peak_ during INC is the “true ⩒o_2max_,” agreement between measurements might simply reflect the fact that participants chose to cease exercise at a similar level of submaximal fatigue (and, by extension, a submaximal ⩒o_2_ that was not significantly different) during both tests. Hence, they suggest that if a severe-intensity CWR bout is performed following INC, the extent of its usefulness is to identify participants who did not achieve their highest ⩒o_2peak_ on INC as opposed to verifying ⩒o_2max_ for individuals who did. In other words, the usefulness of a severe-intensity CWR bout to limit of tolerance following INC might simply be to determine whether a higher ⩒o_2peak_ can be elicited.

One caveat when interpreting the findings presented above regarding verification bouts is that this research was performed on participants who were physically active. The lack of information on sedentary individuals in the literature is perhaps surprising given the prevailing sedentarism and the use of ⩒o_2max_ assessment for individuals who are unaccustomed to exercise (e.g., those that would more likely be found in the clinical setting). Furthermore, the belief that a CWR bout is required to verify ⩒o_2peak_ as ⩒o_2max_ in lieu of a ⩒o_2_ plateau is based on the contention that often-used “secondary criteria” (e.g., threshold values for RER, heart rate, RPE and/or blood lactate concentration) are not valid for this purpose. For example, secondary criteria are satisfied long before T_lim_ when athletes [11] and recreationally-active individuals [12] perform RAMP-INC. However, Misquita et al. (2001) had sedentary overweight/obese postmenopausal women perform two different incremental protocols on a treadmill and concluded that achievement of traditional secondary criteria for HR (220 minus age) and RER (1.10) increases the probability that the highest ⩒o_2_ observed is a true physiological maximum when the majority of participants did not demonstrate a ⩒o_2_ plateau even when a second test was administered [13]. The degree to which traditional secondary criteria are useful might, therefore, depend on the physical-activity and/or obesity status of the individual. Moreover, Wagner et al. have recently proposed new age-dependent secondary criteria for RER and HR based on data collected during RAMP-INC cycling tests performed by a large cohort of healthy men and women aged 20–91 yr. [14]. Importantly, the values they derived, which were calculated using one-sided lower tolerance intervals (confidence level, 95%; coverage, 90%) for tests characterized by a ⩒o_2_ plateau, were higher (RER) and lower (HR) than the aforementioned traditional ones and, therefore, less likely to result in false confirmation (type I error) or false rejection (type II error) of ⩒o_2max_, respectively [14].

The purpose of the present study was to determine the degree to which an additional severe-intensity CWR bout and/or traditional and newly-proposed secondary criteria based on RER and HR might be valid methods to employ when attempting to verify ⩒o_2max_ for sedentary individuals. We did so by comparing the ⩒o_2peak_ during RAMP-INC performed to T_lim_ by sedentary men and women with and without overweight/obesity to the ⩒o_2peak_ during CWR to T_lim_ at 100% of the peak work rate (WR_peak_) achieved on RAMP-INC. Given the previous findings for athletes and recreationally-active individuals (see above), we hypothesized that the ⩒o_2peak_ during CWR would not be significantly different compared to the ⩒o_2peak_ during RAMP-INC. We also hypothesized that traditional criterion values for RER and HR would not be valid for identifying instances when the CWR bout confirmed that RAMP-INC was characterized by a submaximal ⩒o_2peak_. Finally, we hypothesized that newly-proposed age-dependent criterion values for RER and HR [14] would better identify such instances.

## Materials and methods

The data that are presented in this article were collected as part of a cross-sectional trial that included performance of RAMP-INC to T_lim_ followed by CWR at 100% of WR_peak_ on RAMP-INC. The objective was to provide two opportunities for participants to achieve their ⩒o_2max_ (assumed to be the higher of the two values), which was used for group matching for the evaluation that would follow. That evaluation involved a nine-day investigation period which included moderate-intensity CWR bouts to assess the participant’s capacity for lipid oxidation. These bouts were performed at specific percentages of the gas exchange threshold measured during RAMP-INC; however, the ⩒o_2peak_ measured during RAMP-INC was used to ensure that participants were of a similar level of conditioning (≤ average). No other data from the parent study have yet to be published and other than the group mean ± SD for ⩒o_2peak_, none of the data presented in the present article will be included in any other articles derived from the parent investigation when they are published.

### Participants

Data from 35 male (*n* = 19: age, 33.4 ± 6.3 yrs; body mass, 81.7 ± 15.5 kg) and female (age, 26.8 ± 3.6 yrs; body mass, 70.8 ± 14.4 kg) sedentary participants were assessed for this investigation. Participants satisfied criteria for sedentarism based on their responses to two physical-activity questionnaires. The Paffenbarger Physical Activity Questionnaire (PPAQ) is a self-administered survey with eight questions designed to quantify participation in leisure-time physical activity for young and older adults [15]. The first four questions inquire as to the number of city blocks participants typically walked, the flights of stairs they typically climbed each day and the frequency and duration of sport and recreational activities that they had performed over the past year [16]. A physical activity index is calculated from the answers to these questions. The International Physical Activity Questionnaire (IPAQ) is a survey that assesses sedentary behavior that can be administered in person or over the phone. Participants are asked to list the time they spend sitting throughout their day including at work, at home, in a motor vehicle, while doing course work and during leisure time activities (e.g., watching television). The IPAQ has been shown to possess adequate reliability and validity for assessing sedentary behavior for women and men from multiple countries [17].

Participants in our study were healthy; however, owing to the methodology of the parent study, both normal weight and overweight/obese individuals (body mass index, 19.0–24.9 and 25.0–35.0 kg∙m^-2^, respectively) were recruited. Table 1 provides the breakdown of our participant population for both body fatness and sex. Exclusion criteria that were established based on the requirements of the parent study included: 1.) known cardiovascular, pulmonary and/or metabolic disease; 2.) medication intake that would affect the metabolic response to exercise; 3.) smoking within the past six months; 4.) ethanol intake > 2 oz per day; 5.) weight change ≥ 3 kg within the past three months; and 6.) ⩒o_2max_ > the age-/gender-defined average [18]. Participants provided written informed consent to take part in the parent study, which was approved by the Institutional Review Board of the ICAHN School of Medicine at Mount Sinai Hospital. In addition to RAMP-INC, the informed consent form that was approved by the IRB included an explanation of the severe-intensity CWR bout.

**Table 1:** Sex distribution of participants who were classified according to BMI as normal weight, overweight or obese in the present study (total *n* = 35).

	Normal Weight (*n*)	Overweight (*n*)	Obese (*n*)
	(19.0–24.9 kg∙m^-2^)	(25.0–29.9 kg∙m^-2^)	(30.0–34.9 kg∙m^-2^)
Male	9	7	3
Female	8	1	7

### Exercise testing

After initial telephone screening, prospective participants visited the laboratory for a comprehensive medical examination that included blood work, an ECG and an oral glucose tolerance test. Following this screening session, individuals deemed appropriate for inclusion returned to the laboratory on another day for the final stage of pre-trial screening before beginning the nine-day parent study. This visit occurred in the morning with participants in the fasted state having refrained from consuming calorie-containing foods and beverages for 12 hrs. Participants were also instructed to refrain from ingesting any product containing caffeine and/or alcohol for this 12-hr period and to avoid moderate or strenuous activity for the prior 24 hrs. The first test during this visit required participants to lie supine on a bed with a hood placed over their head so that gas exchange and ventilation could be measured for 60 min. The purpose was to determine the participant’s resting metabolic rate. Following this procedure and while still in the fasted state, participants performed the two exercise bouts from which the data presented in this article were derived. The two-test sequence was performed on an electronically-braked cycle ergometer (VIAsprint 150P, Ergoline, Bitz, Germany). The RAMP-INC began with 4 min of unloaded “baseline” cycling followed by an increase in work rate of 1 W every 3 or 4 s for male and female participants, respectively. Participants maintained a pedal cadence of 60 ± 2 rpm during both cycling bouts and the tests were terminated when this cadence was unable to be maintained for ≥ 10 s despite strong verbal encouragement. Verbal encouragement throughout the tests was based on the 20-s protocol advanced by Andreacci et al. (2002) [19]. Once RAMP-INC was terminated, participants performed cool-down cycling at 25 W for 10 min after which they rested on the ergometer for a 2–3 min period that was required for the CWR work rate to be programmed. Participants then resumed pedaling at 100% of the WR_peak_ from RAMP-INC and continued to do so until they reached T_lim_. The cadence and termination criterion for the CWR bout was the same as that which was used for RAMP-INC (see above).

During both exercise tests, participants breathed through a low dead-space mouthpiece so that gas-exchange and ventilation data could be collected breath by breath (Carefusion Vmax Encore, Yorba Linda, CA). The gas analyzers were calibrated with gases of known concentration prior to each testing session and the flow sensor was calibrated using a 3-L syringe (Hans Rudolph Inc.). Blood pressure (SunTech Tango M2, Morrisville, NC), HR and ECG were also monitored continuously during the tests. The WR_peak_ and time to T_lim_ on RAMP-INC were recorded and participants rated their whole-body sense of effort at T_lim_ using the Borg RPE scale (6–20) immediately following the test. Time to T_lim_ and RPE were also recorded for CWR.

### Data analysis

To compare peak values for ⩒o_2_, RER and HR between INC and CWR, gas exchange and ventilation data were exported in 10-s bins that were subsequently rolling averaged to provide 20-s values. The peak values for each variable were defined as the highest 20-s rolling-average value measured during each test. To evaluate secondary criteria that have traditionally been used for verifying ⩒o_2max_ during INC, we determined the number of participants who achieved/surpassed an RER of 1.10 and/or a HR of 95% of the age-predicted maximum (220 minus age) on RAMP-INC. To evaluate newly-proposed age-dependent criteria, we determined the number of participants who achieved/surpassed an RER of 1.13, a HR of 96% of the adjusted age-predicted maximum (210 minus age; APMHR_210_) and/or 93% of the age-predicted maximum based on the formula recommended by Tanaka et al. (208 minus 0.7*age; APMHR_208_) for aged 20–39 yr or achieved/surpassed an RER of 1.10, a HR of 99% of APMHR_210_ and/or 92% of APMHR_208_ for aged 40–59 yr [14]. In each case, for these participants, we also determined the ⩒o_2_ values that were present at the point at which the RER and HR criterion values were achieved during the test and then expressed them as a percentage of the ⩒o_2peak_ during RAMP-INC and the higher ⩒o_2peak_ achieved across both tests. Finally, to test for the presence of a ⩒o_2_ plateau during the final portion of RAMP-INC, linear regression was used to predict the ⩒o_2_ values during the final 120 s of exercise. For this analysis, data were averaged into 20-s bins and the fitting window was constrained to exclude the initial and final 120 s of exercise to ensure that the fit was not contaminated by the initial lag in the ⩒o_2_ response during RAMP-INC (i.e., the ⩒o_2_ mean response time) [4] and any leveling off that did occur prior to T_lim_. The slope of this relationship was then used to predict 20-s changes that should have been present had the response maintained the same trajectory throughout the final 120 s. Specifically, any of the six values lying below the lower boundary of the 95% confidence interval associated with estimation of the slope parameter [7] were flagged based on the possibility that such values might represent a leveling off of the ⩒o_2_ response in comparison to the relationship that was present throughout the test. However, given the inherent “noise” in the pulmonary gas-exchange signal, we visually inspected all flagged values to determine whether a discernible leveling off was indeed present. Furthermore, the linear fit for each participant was extrapolated through the final 120 s of exercise so that residuals (observed–predicted values) could be determined. A tendency for negative residuals also resulted in the response being flagged for visual inspection to determine whether a ⩒o_2_ plateau was present.

### Statistical analysis

All statistical analyses were performed using SPSS version 23 (SPSS, Chicago, IL). Data are presented as means ± SD. Within-subject comparisons were made using paired *t*-tests. The slope and *y*-intercept of the ⩒o_2_-work rate response during RAMP-INC were determined by using standard least-squares regression analysis. Correlations were assessed the same way. In addition to an association between the ⩒o_2peak_ values for the two bouts, we tested for correlations between the difference in ⩒o_2peak_ for the two bouts and WR_peak_ on RAMP-INC, T_lim_ on RAMP-INC, T_lim_ on CWR and the higher of the two ⩒o_2peak_ values achieved across the tests. Bland-Altman plots and a one-sample *z*-test [20] were used to investigate average bias, precision and limits of agreement between ⩒o_2peak_ measurements from the two tests [10]. For all analyses, significance was accepted at *p* < 0.05.

## Results and discussion

**Fig 1:**
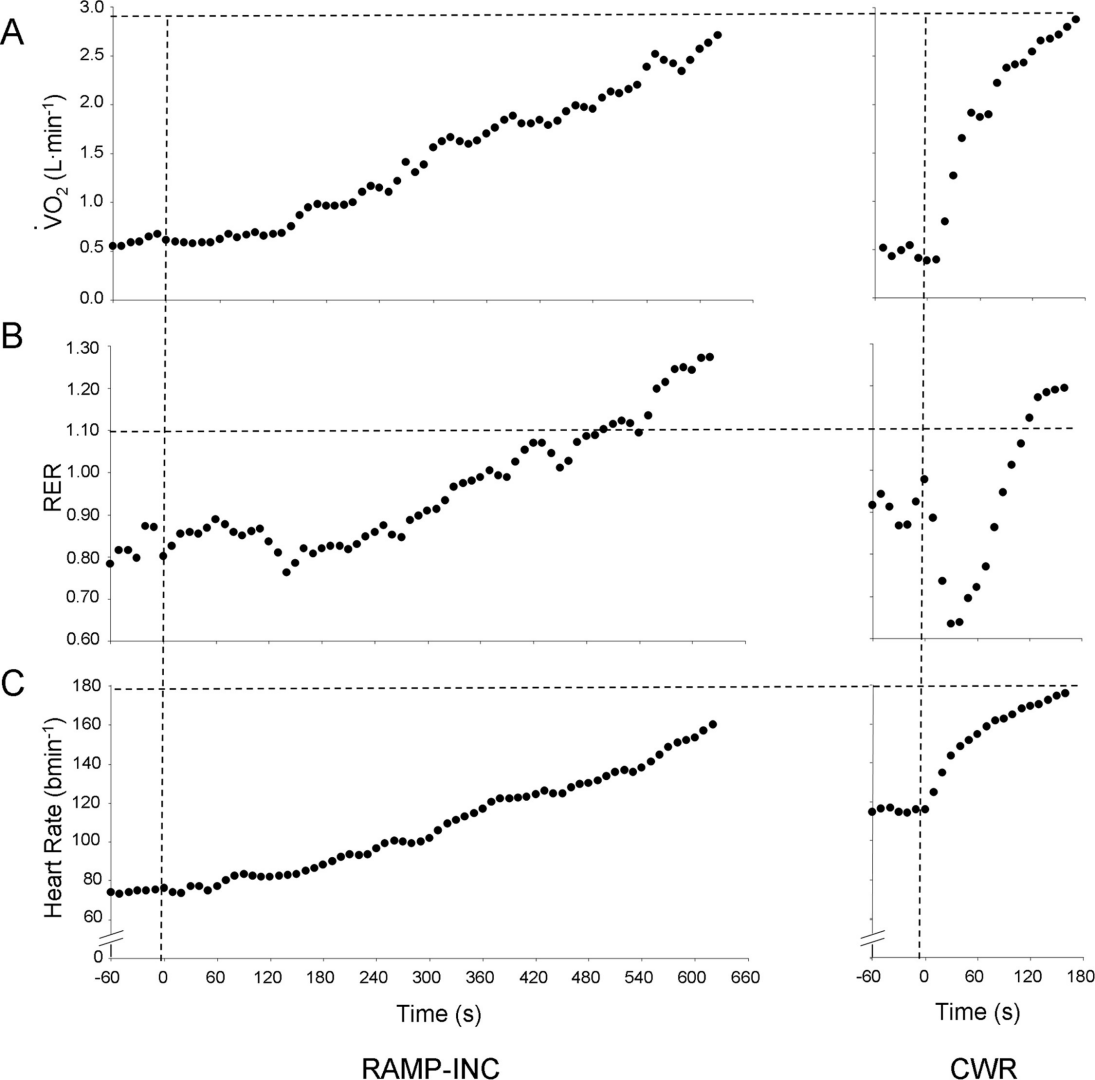
Representative-subject data for ⩒o_**2**_ (Panel A), RER (Panel B) and HR (Panel C) for the two tests. Vertical dashed lines represent the onset of exercise for RAMP-INC (left side) and CWR (right side). Horizontal dashed lines indicate the higher ⩒o_**2peak**_ observed across the two tests (Panel A) and traditional criterion values for RER (1.10; Panel B) and HR (95% of the age-predicted maximum; Panel C) that are often used to verify attainment of ⩒o_**2max**_.

**Fig 2:**
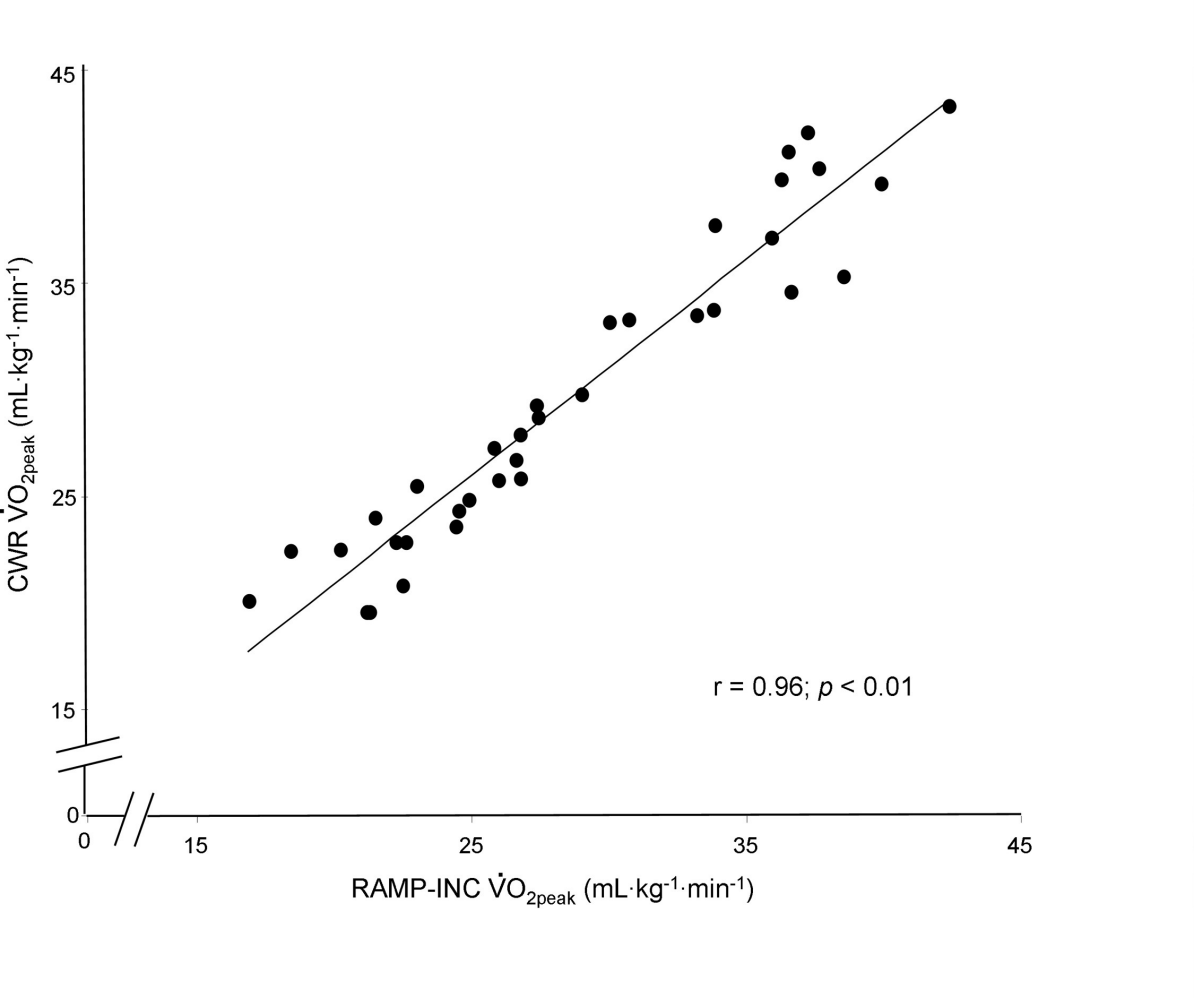
Relationship between the ⩒o_2peak_ values for RAMP-INC and CWR. A significant correlation was observed for the measurements derived from the two tests.

**Fig 3:**
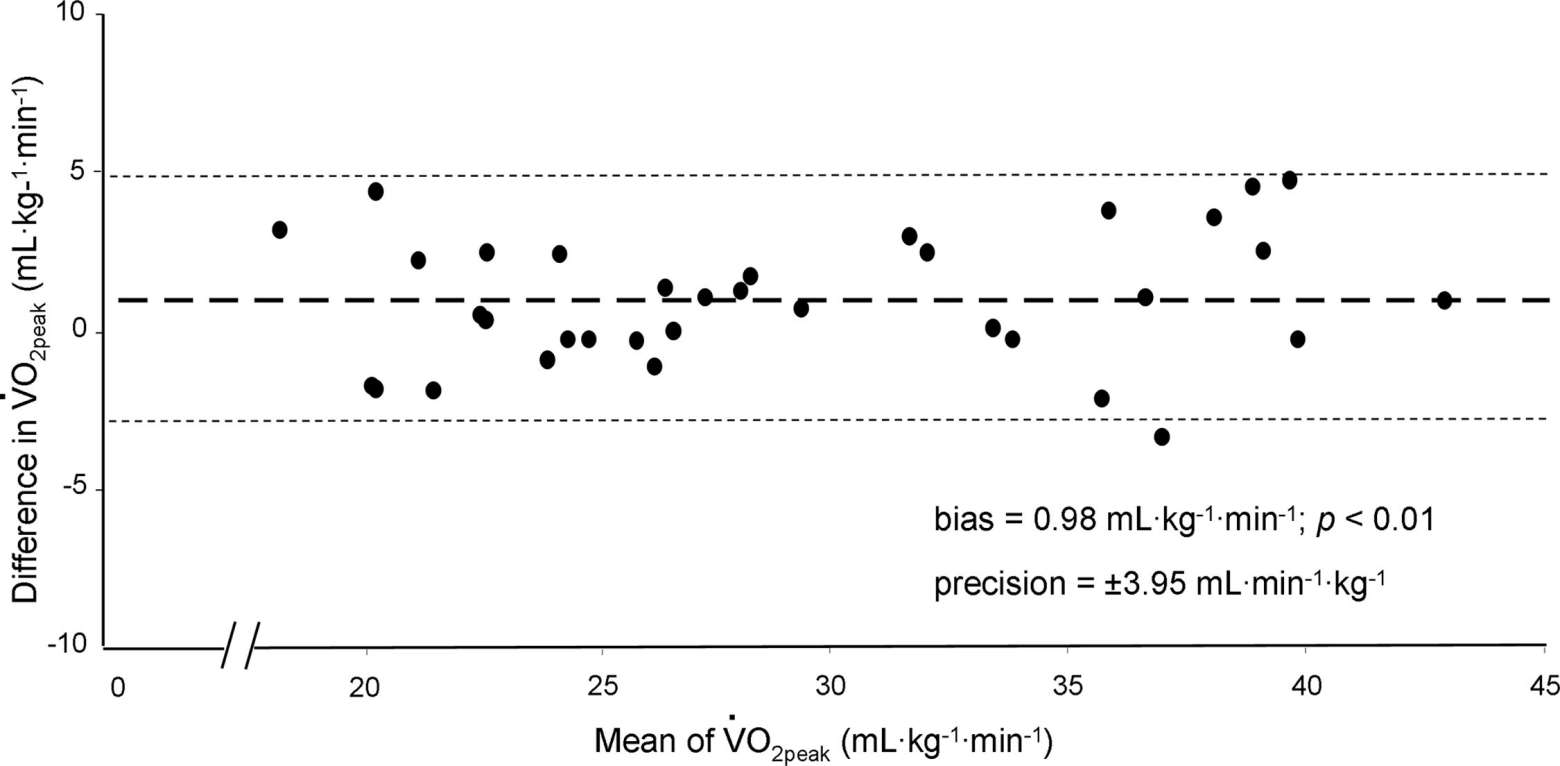
Bland-Altman plot depicting individual-subject absolute differences between measurements of ⩒o_2peak_ for RAMP-INC and CWR as a function of the average of the two measurements. Bold dashed line and fine dashed lines represent bias and precision, respectively.

Representative-subject data for the ⩒o_2_ and HR responses during RAMP-INC and CWR are depicted in Fig 1 and the peak ⩒o_2_, RER, HR and RPE during the two tests are provided in Table 2. The WR_peak_ achieved on RAMP-INC and, therefore, the work rate maintained during CWR was 174 ± 41 W (range, 103–275 W). The time to T_lim_ was 574 ± 106 s (range, 410–780 s) and 155 ± 32 s (range, 100–240 s) for RAMP-INC and CWR, respectively. The ⩒o_2peak_ values for the two bouts were highly correlated (*r* = 0.96; *p* < 0.01; mean CV = 24%) (Fig 2); however, the ⩒o_2peak_ for CWR was significantly greater than the ⩒o_2peak_ for RAMP-INC (Table 1). In this regard, a mean bias of 0.98 mL∙min^-1^∙kg^-1^ that was significantly different from zero (*z* = -2.9, *p* < 0.01) with a precision of ± 3.95 mL∙min^-1^∙kg^-1^ was observed (Fig 3). Specifically, 23 of 35 participants achieved a higher ⩒o_2peak_ on CWR compared to RAMP-INC (range, 0.04–4.70 mL∙kg^-1^∙min^-1^, 0.1–23.7% of the RAMP-INC value). Moreover, for 16 of these 23 individuals, the difference exceeded 3%, the biological variability that is inherent with ⩒o_2max_ measurement [7]. The difference between the ⩒o_2peak_ for CWR compared to RAMP-INC was not significantly correlated with WR_peak_ on RAMP-INC (*r* = 0.13), T_lim_ on RAMP-INC (*r* = 0.04) or CWR (*r* = 0.41) or the higher of ⩒o_2peak_ values achieved across the two tests (*r* = 0.25) (*p* > 0.05 in all cases). The RER_peak_ was significantly greater for RAMP-INC compared to CWR whereas HR_peak_ and end-exercise RPE were not significantly different between tests (Table 2).

**Table 2:** Peak values during ramp incremental test and a constant-work-rate bout performed at the highest work rate achieved on the ramp test for sedentary individuals (*n* = 35) in the present study.

	RAMP-INC	CWR	*p*
⩒o_2peak_ (L∙min^-1^)	2.18 ± 0.61	2.26 ± 0.65*	.004
⩒o_2peak_ (mL∙min^-1^∙kg^-1^)	28.6 ± 6.8	29.6 ± 7.2*	.007
RER_peak_	1.26 ± 0.10	1.20 ± 0.08*	.000
HR_peak_ (beats∙min^-1^)	170 ± 12	172 ± 9	.133
End-exercise RPE	18.2 ± 1.2	18.3 ± 1.3	.518

Data are presented as the mean ± SD. ⩒o_2peak_, peak rate of oxygen uptake; RER_peak_, peak respiratory exchange ratio; HR_peak_, peak heart rate; RPE, rating of perceived exertion.

*, Significant difference from RAMP-INC (*p* < 0.05).

An RER ≥ 1.10 (traditional criterion) was achieved on RAMP-INC by 33 of 35 participants including 21 of the 23 that were capable of achieving a higher ⩒o_2peak_ on CWR compared to RAMP-INC. For participants who satisfied the traditional RER criterion on RAMP-INC, the concurrent ⩒o_2_ value when it was reached was 75 ± 12% and 72 ± 11% of the ⩒o_2peak_ attained on RAMP-INC and across both tests, respectively. An RER ≥ 1.13 (aged 20–39 yrs; *n* = 31) or 1.10 (aged 40–59 yrs; *n* = 4) (newly-proposed age-dependent criterion) was achieved by the same 33 of 35 participants although in this case, the concurrent ⩒o_2_ value when the newly-proposed age-dependent criterion was reached was 80 ± 13% and 76 ± 12% of the ⩒o_2peak_ attained on RAMP-INC and across both tests, respectively. A HR of ≥ 95% of the age-predicted maximum (220 –age) (traditional criterion) was achieved by seven of 35 participants including 3 of the 23 that were capable of achieving a higher ⩒o_2peak_ on CWR compared to RAMP-INC. For participants who satisfied the traditional HR criterion, the concurrent ⩒o_2_ value when it was reached was 93 ± 7% and 91 ± 7% of the peak value attained on RAMP-INC and across both tests, respectively. A HR ≥ 96% (aged 20–39 yrs) or 99% (aged 40–59 yrs) of APMHR_210_ (one of the newly-proposed age-dependent criterion) or 93% (aged 20–39 yrs) or 92% (aged 40–59 yrs) of APMHR_208_ (the other newly-proposed age-dependent criterion) was achieved by 14 of 35 participants (i.e., the seven participants who achieved the traditional criterion plus seven others) including 8 of the 23 that were capable of achieving a higher ⩒o_2peak_ on CWR compared to RAMP-INC. For participants who satisfied the newly-proposed age-dependent criterion based on APMHR_210_, the concurrent ⩒o_2_ value when it was reached was 90 ± 8% and 87 ± 9% of the peak value attained on RAMP-INC and across both tests, respectively. For participants who satisfied the newly-proposed age-dependent criterion based on APMHR_208_, the concurrent ⩒o_2_ value when it was reached was 91 ± 7% and 87 ± 7% of the peak value attained on RAMP-INC and across both tests, respectively.

The group mean ± SD for the slope and *y*-intercept of the ⩒o_2_-work rate response during RAMP-INC were 10.7 ± 1.4 mL∙min^-1^∙W^-1^ and 0.65 ± 0.11 mL∙min^-1^, respectively (*r* = 0.97 ± 0.04; range, 0.83–1.00). When the slope and intercept values for each participant were used to predict their ⩒o_2_ values for the final two minutes of exercise during RAMP-INC (i.e., the final six 20-s values), a ⩒o_2_ plateau was deemed present for two of 35 participants. During RAMP-INC, both of these participants surpassed the traditional criterion value for RER; however, neither surpassed the traditional criterion value for HR. Both of these participants achieved a higher ⩒o_2peak_ (6.3% and 11.0%) on CWR despite the apparent plateau during RAMP-INC.

The main finding from this study is that a CWR cycling bout to limit of tolerance at the WR_peak_ from a RAMP-INC performed 10 min prior resulted in a significantly higher ⩒o_2peak_ compared to that which was achieved on RAMP-INC. This refuted our first hypothesis and indicates that RAMP-INC underestimates ⩒o_2max_ in a heterogeneous population of sedentary individuals. Our second hypothesis was partially supported as the traditional criterion value for RER was not valid for identifying instances when the CWR bout confirmed that RAMP-INC was characterized by a submaximal ⩒o_2peak_. However, despite the fact that it was surpassed on average at ~93% of the bout’s ⩒o_2peak_, the fact that the criterion value for HR identified 20 of the 23 individuals who did not achieve their ⩒o_2max_ on RAMP-INC suggests that at least for some individuals, it might have been valid for this purpose. Finally, contrary to our third hypothesis, use of newly-proposed age-dependent values for RER and HR [14] did not improve the ability to identify instances when CWR confirmed that RAMP-INC was characterized by a submaximal ⩒o_2peak_. Specifically, use of more stringent RER values yielded the same conclusion (acceptance of ⩒o_2max_ for 33 of 35 RAMP-INC tests including 23 during which it was confirmed to have not been reached) while use of less stringent HR values resulted in seven additional confirmations of ⩒o_2max_ on RAMP-INC that included five tests during which the CWR bout refuted that conclusion.

In the present study, we found that participants achieved a significantly higher ⩒o_2peak_ during CWR cycling in the severe-intensity domain compared to that which they achieved during a RAMP-INC cycling test when both bouts were performed to the limit of exercise tolerance. However, given inherent biological variability in the measurement of ⩒o_2max_, using grouped data (i.e., in this case, a significant difference between group means) to support such a difference (or lack thereof) has been criticized [21]. Hence, to further investigate the difference that was observed between measurements from the two tests, we examined limits of agreement between measurements and individual-subject data. In the present study, a positive bias was present (0.98 mL∙min^-1^∙kg^-1^ or 3.4% of the RAMP-INC value; see Fig 3) with 23 of 35 participants registering a higher ⩒o_2peak_ on CWR. Importantly, 16 of these 23 demonstrated a value that was ≥ 3% higher, which should account for the influence of biological variability that is inherent with ⩒o_2max_ measurements [7]. However, seven participants registered a ⩒o_2peak_ that was ≥ 3% lower on the CWR, which is important to consider because for these individuals, the additional test was not necessary to provide a more accurate measurement. Collectively, we believe these findings support our contention that ⩒o_2max_ is not consistently achieved during RAMP-INC across a heterogeneous (i.e., with respect to sex and body composition) population of sedentary participants.

Our finding that the ⩒o_2peak_ during CWR exceeded the ⩒o_2peak_ during RAMP-INC (*p =* 0.04) by an amount that was greater than the normal variation associated with ⩒o_2max_ measurement for 16 of 35 individuals contrasts with a recent report by Murias et al. who found that CWR cycling bouts at either 85% or 105% of WR_peak_ resulted in ⩒o_2peak_ values that were not significantly different from the ⩒o_2peak_ measured on RAMP-INC [10]. Indeed, in that study, a mean difference between measurements that was not significantly different from zero was observed with individual-subject data revealing only six of 61 participants that achieved a ⩒o_2peak_ that was “higher” (i.e., ≥ 2 ml∙kg^-1^∙min^-1^ greater) than the RAMP-INC value on the CWR bout [10]. There are a number of differences between our study and the study of Murias et al. that might explain these contrasting findings. The major one is that unlike the “recreationally-active” participants recruited in that investigation, our participants were sedentary (e.g., RAMP-INC ⩒o_2peak_, 28.6 ± 6.8 mL∙min^-1^∙kg^-1^ vs. 39.8 ± 11.5) [10]. This is relevant because individuals with a lower level of conditioning and/or less familiarity with physical activity might be more apt to terminate RAMP-INC performed to “limit of tolerance” based on factors other than exhaustion of the physiological factor(s) that rate limit(s) ⩒o_2max_ in more conditioned individuals; i.e., the integrative function of the pulmonary, cardiovascular and muscular systems to uptake, transport and utilize O_2_ [22]. For example, local muscular fatigue, lack of motivation, boredom and the discomfort associated with sitting on the cycle seat and breathing with gas-exchange collection might all be more apt to present a limitation during a cycling bout lasting ~10 min for individuals less accustomed to exercise. Conversely, severe-intensity CWR initiated after a short rest period following RAMP-INC might be more apt to remove such a limitation because work rate begins at a high level and metabolism is already elevated at exercise onset. Collectively, these differences mean that for severe-intensity CWR like that which we employed in this study, less time is required for ⩒o_2_ to reach and possibly exceed the highest value observed during RAMP-INC if RAMP-INC was terminated prematurely.

Exercise performed at a constant rate of work within the severe-intensity domain (i.e., at work rates situated above the asymptote of the hyperbolic power/T_lim_ relationship, but below the point at which that hyperbola converges with the one that describes the power/time-to-⩒o_2max_ relationship; i.e., work rates that are above the critical power, but do not fall within the “extreme” domain which exceeds the severe domain) results in the achievement of ⩒o_2max_ if the bout is continued until T_lim_ [6, 9]. This forms the basis for the suggestion that a severe-intensity bout performed at a work rate that exceeds WR_peak_ achieved on RAMP-INC can be used to test for a “⩒o_2_ plateau” that verifies the value as ⩒o_2max_ in lieu of an observed plateau during the final portion of RAMP-INC [7]. However, in our study, due to concerns regarding the ability of sedentary individuals to maintain supramaximal work for a long enough period of time for ⩒o_2max_ to be reached, we had participants perform CWR at a work rate that was only equal to the WR_peak_ achieved during RAMP-INC (see below). Consequently, even when the “same” ⩒o_2peak_ was observed for the two tests, we could not confirm a plateau (i.e., a similar ⩒o_2_ response for a greater rate of work) across tests. Furthermore, even in cases where a supramaximal work rate is maintained during CWR, the fact that the work rate is endured during a separate bout (as opposed to one continuous one where work rate is increased throughout) means that it is conceivable that participants could simply stop exercising at the same submaximal ⩒o_2_ during both tests [10]. For these reasons, we are hesitant to refer to the bout we employed as a “verification bout.” However, contrary to recent suggestions [10], at least for the type of individual we assessed, the addition of a severe-intensity CWR bout following RAMP-INC was necessary because it resulted in a second opportunity for participants to achieve, if not their ⩒o_2max_, at least a value that was situated closer to it. This was important because we used this measurement to verify the sedentary status of our participants and to match the various groups that we were stratifying for comparison in our parent study.

In addition to findings from studies of athletes [11, 23] and non-sedentary individuals [5, 10, 12, 24, 25], our observation that the ⩒o_2peak_ during a severe-intensity CWR to limit of tolerance is significantly greater than the ⩒o_2peak_ during a prior RAMP-INC contrasts findings from a number of investigations of individuals with similar characteristics as those that comprised our cohort. For example, Sawyer et al. investigated sedentary individuals with obesity and found no significant difference between the ⩒o_2peak_ during RAMP-INC and that which they measured during a CWR bout like the one we employed (i.e., performed 5–10 min later at 100% WR_peak_) [26]. However, in that study, 13 of 19 participants achieved a value during CWR that was ≥ 2% higher (range, 2.0–21.0%; 0.04–0.47 L∙min^-1^) [26]. Astorino et al. performed two different assessments of sedentary individuals and found no significant difference between the ⩒o_2peak_ values for RAMP-INC and CWR [27]. However, these researchers used supramaximal work rates for CWR bouts performed long enough after RAMP-INC to allow ⩒o_2_ to return to its resting level; specifically, 105% of WR_peak_ after 24 hrs (study 1) and 115% of WR_peak_ after 60–90 min (study 2) [27]. These differences might explain the contrasting findings compared to our study. Sedentary individuals would likely possess slower ⩒o_2_ kinetics which would result in a higher proportion of “non-sustainable” work rates (i.e., work rates situated above the “critical power”) that they would not be able to maintain long enough to reach ⩒o_2max_ (i.e., work rates situated within the extreme-intensity domain). In conjunction with removal of the ⩒o_2_ increase consequent to the elevated baseline metabolic rate (e.g., in our study attributable to CWR being performed only ~10 min after completion of RAMP-INC), this might have resulted in CWR work rates that were too high to elicit a large enough increase to reach ⩒o_2max_ (or at least a ⩒o_2peak_ that was greater than that which was achieved during RAMP-INC) in those studies.

Consistent with previous findings on recreationally-active [5, 8] and sedentary [13, 28] individuals, a ⩒o_2_ plateau was not a consistent feature at T_lim_ during RAMP-INC in the present study. Indeed, using objectively-derived criteria based on the ⩒o_2_-WR slope (as opposed to the lack of an arbitrarily-determined absolute generic increase), we identified only two individuals who demonstrated a plateau during RAMP-INC. In lieu of identification of such a plateau, several criteria have been suggested to be useful for verifying that ⩒o_2max_ has been reached. Being that we were able to identify RAMP-INC tests characterized by a submaximal ⩒o_2peak_, our methodology allowed us to assess the degree to which often-used traditional and newly-proposed age-dependent [14] values for RER and HR are useful for this purpose. In agreement with the first part of our second hypothesis, the inability for participants to achieve the traditional criterion value of 1.10 for RER was not a consistent feature of RAMP-INC tests characterized by a submaximal ⩒o_2peak_. Indeed, the value was surpassed during 21 of the 23 RAMP-INC bouts that were characterized by a lower ⩒o_2peak_ compared to CWR including 14 of the 16 during which the value was ≥ 3% lower. However, contrary to the first part of our third hypothesis, the newly-proposed age-dependent criterion was equally as ineffective as all participants aged 20–39 yrs (*n* = 31) that exceeded 1.10 (*n* = 29) also exceeded 1.13 (i.e., the newly-proposed value for that age group). The newly-proposed value for individuals aged 40–59 was also 1.10 so additional analysis was not necessary.

Unlike an RER of 1.10 or 1.13, which was “easy” for participants to achieve (e.g., achieved at ~72% and ~76% of the highest ⩒o_2peak_ attained across both tests, respectively), the criterion values for HR that we used in this study were more difficult for participants to surpass. For example, only three of the 23 individuals who achieved a higher ⩒o_2peak_ on CWR compared to RAMP-INC achieved a HR during RAMP-INC that exceeded 95% of their age-predicted maximum (220 minus age), which was the traditional criterion that we employed. This supports the contention that achievement of a threshold level for HR might provide a useful way to identify RAMP-INC tests characterized by a submaximal ⩒o_2peak_ for sedentary individuals. However, it is important to note that only four of the 12 participants who did not achieve a greater ⩒o_2peak_ during CWR were able to satisfy this criterion during RAMP-INC. While we cannot confirm that the RAMP-INC ⩒o_2peak_ was ⩒o_2max_ for these individuals (see above), it seems unlikely that it definitely was not for eight of the 12. Furthermore, this criterion HR value was equally difficult to achieve during CWR as only six of the 35 participants (all of whom also achieved it on RAMP-INC) were able to do so. This means that if this particular HR value was valid as a threshold indicator of the attainment of ⩒o_2max_ (i.e., did not result in a high proportion of rejection of tests during which it actually was achieved), 29 of 35 participants in our study would not have achieved their ⩒o_2max_ on either test. This seems unlikely, which implies that this value for HR is too stringent to be used for accepting that ⩒o_2max_ has been achieved with this type of individual. Furthermore, even if it did indicate submaximal effort at the limit of tolerance during both tests, it does not refute the use of the additional test because a high percentage of participants would have then achieved a submaximal ⩒o_2_ that was at least closer to their maximum value upon completion of the CWR bout. Use of the newly-proposed criteria for HR based on either APHRM_210_ or APHRM_208_ [14] resulted in acceptance of an additional seven tests, five of which were characterized by a lower ⩒o_2peak_ compared to CWR. This means that compared to use of the traditional HR criterion, use of either of the less stringent newly-proposed ones resulted in acceptance of ⩒o_2max_ for two additional RAMP-INC tests during which that might have been the case at the expense of acceptance of ⩒o_2max_ for five additional tests during which it definitely was not.

There are limitations with the present study that deserve recognition. As previously mentioned, we chose a severe-intensity work rate for the CWR bout that was equal to the highest attained on the RAMP-INC. Hence, our protocol did not conform to the one that has been advanced for verification bouts which involve supramaximal work rates designed to test whether ⩒o_2_ can be driven to a higher value than that which was observed on RAMP-INC [7]. However, being that we found that in 23 of 35 cases, ⩒o_2_ could be driven to a higher value during CWR even if it is performed at only the maximal work rate, it is intuitive that the same result would have been present if we had used a supramaximal work rate assuming it did not result in an “extreme” as opposed to severe domain-specific response. Nevertheless, future research involving post-INC CWR bouts at supramaximal work rates (e.g., 105% of WR_peak_) and, perhaps, submaximal work rates that allow for longer duration CWR (e.g., 95% WR_peak_) as well as multiple bouts to assess test-retest reliability will be important for providing more insight. Another limitation is that owing to the requirements of the parent study, participants performed the two-test sequence in a fasted state having refrained from consuming calorie-containing foods and beverages for 12 hrs. Consequently, exercise performance (and the ability to achieve a “true ⩒o_2max_”) might have been compromised by the lack of pre-test feeding during both tests. Interestingly, previous research indicates that overnight fasting does not impair the peak ⩒o_2_ response for competitive cyclists [29]; however, the degree to which this is also the case for sedentary individuals like the ones that we assessed requires further investigation. Finally, it is important to note that our methodology did not provide an opportunity to familiarize participants with the performance of exhaustive cycle-ergometer exercise prior to testing. Consequently, individuals like these who are unaccustomed to exercise in general might have reached a higher ⩒o_2peak_ during the second of two such bouts regardless of the type of exhaustive exercise that was performed. Ultimately, the cost-benefit ratio of using the additional CWR test in the research setting must be considered to determine whether it is warranted to include in order to identify what we observed; specifically, a significant difference in ⩒o_2peak_ response with CWR registering a greater value for 66% of sedentary individuals with the difference exceeding normal biologic variability in 70% of those cases.

## Conclusions

In conclusion, a severe-intensity CWR bout to limit of tolerance at the highest work rate achieved during a RAMP-INC test concluded 10 minutes prior resulted in a significantly higher ⩒o_2peak_ compared to that which was reached on RAMP-INC for sedentary individuals. While we cannot confirm that CWR revealed the ⩒o_2max_ in these cases, our findings suggest that giving this type of individual a second chance to achieve their highest ⩒o_2_ response during a subsequent CWR test that is ~70% shorter than RAMP-INC increases the likelihood that they will register a value that, if not their ⩒o_2max_, is at least closer to it. The popular and often-advocated criterion value of 1.10 for RER was too lenient for identifying RAMP-INC tests characterized by a submaximal ⩒o_2peak_ as was the newly-proposed age-dependent value of 1.13. Conversely, the HR value of 95% of the age-predicted maximum appeared to be too stringent with the newly-proposed ones potentially reducing the chance of type II error at the expense of a greater likelihood of false acceptance.

## Supporting information

S1 Table Raw data. (XLS)
